# Therapeutic application of carvacrol: A comprehensive review

**DOI:** 10.1002/fsn3.2994

**Published:** 2022-08-03

**Authors:** Muhammad Imran, Mahwish Aslam, Suliman A. Alsagaby, Farhan Saeed, Ishtiaque Ahmad, Muhamamd Afzaal, Muhammad Umair Arshad, Mohamed A. Abdelgawad, Ahmed H. El‐Ghorab, Ahmed Khames, Mohammad Ali Shariati, Arslan Ahmad, Muzamal Hussain, Ali Imran, Saiful Islam

**Affiliations:** ^1^ Department of Food Science and Technology University of Narowal Narowal Pakistan; ^2^ Faculty of Allied Health Sciences, University Institute of Diet and Nutritional Sciences The University of Lahore Lahore Pakistan; ^3^ Department of Medical Laboratory Sciences, College of Applied Medical Sciences Majmaah University Majmaah Saudi Arabia; ^4^ Department of Food Science and Technology Government College University Faisalabad Pakistan; ^5^ Department of Dairy Technology, FAPT University of Veterinary & Animal Sciences Lahore Pakistan; ^6^ Department of Pharmaceutical Chemistry, College of Pharmacy Jouf University Sakaka Saudi Arabia; ^7^ Department of Chemistry, College of Science Jouf University Sakaka Saudi Arabia; ^8^ Department of Pharmaceutics and Industrial Pharmacy, College of Pharmacy Taif University Taif Saudi Arabia; ^9^ K.G. Razumovsky Moscow State University of Technologies and Management (The First Cossack University) Moscow Russian Federation; ^10^ Institute of Nutrition and Food Science University of Dhaka Dhaka Bangladesh

**Keywords:** anticancer, antidiabetic, cardio‐protective, carvacrol, dose and safety, phytochemical

## Abstract

Carvacrol is a major natural constituent and is significantly present as an essential oil in aromatic plants and is well known for its numerous biological activities. Therapeutic properties of carvacrol have been demonstrated as anti‐oxidant, anticancer, diabetes prevention, cardioprotective, anti‐obesity, hepatoprotective and reproductive role, antiaging, antimicrobial, and immunomodulatory properties. The carvacrol biosynthesis has been mediated through mevalonate pathway. Carvacrol has the anticancer ability against malignant cells via decreasing the expressions of matrix metalloprotease 2 and 9, inducing apoptosis, enhancing the expression of pro‐apoptotic proteins, disrupting mitochondrial membrane, suppressing extracellular signal‐regulated kinase 1/2 mitogen‐activated protein kinase signal transduction, and also decreasing the phosphoinositide 3‐kinase/protein kinase B. It also decreased the concentrations of alanine aminotransferase, alkaline phosphatase and aspartate aminotransferase, and gamma‐glutamyl transpeptidase as well as also restored liver function, insulin level, and plasma glucose level. Carvacrol also has been found to exert antimicrobial activity against *Staphylococcus aureus*, *Pseudomonas aeruginosa*, *Coagulase‐negative staphylococcus*, *Salmonella* spp*.*, *Enterococcus* sp. *Shigella*, and *Escherichia coli*. The current review article summarizes the health‐promoting perspectives of carvacrol through various pathways.

## Graphical abstract

Carvacrol is a major natural constituent in applauded for their nutraceutical potential ranging from antioxidant to anticancer

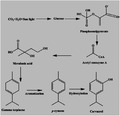

## 1 INTRODUCTION

Carvacrol (2‐Methyl‐5‐[1‐methyl ethyl]‐phenol) is a naturally occurring phenolic monoterpenoid and cymene derivative. Its chemical formula is C_6_H_3_CH_3_ (OH) (C_3_H_7_) and is naturally present in thyme (*Thymus vulgaris*), wild bergamot (*bergamia Loise var. Citrus aurantium*), *Origanum scabrum*, *black cumin*, *Origanum microphyllum*, *Origanum onites*, oregano (*Origanum vulgare*), and pepperwort (*Lepidium flavum*). Carvacrol oil extracted from thyme is 5%–75%, whereas 50%–70% oil is extracted from the marjoram and hop marjoram (Ares et al., 2020; Churklam et al., 2020; Lee et al., 2020; Sun et al., 2020; Tampau et al., 2020).

Its boiling point is 237–238°C (lit.) and it melts at 1°C (lit.). The density of carvacrol is ranged from 0.976 g/cm^3^ at 20°C to 0.975 g/cm^3^ at 25°C. It is not soluble in water but highly soluble in ethanol, carbon tetrachloride, and diethyl ether (Alagawany et al., 2015; Churklam et al., 2020; Lee et al., 2020; Mousavi et al., 2020). Biological activities of carvacrol have been shown in different in vivo and in vitro studies including anti‐oxidant, antiseptic, anticarcinogenic, anti‐inflammatory, antidiabetes role, immunomodulatory, antimicrobial activity, antispasmodic, antibacterial, and growth promoter. As it is a natural cymene derivative, it has potent bacterial inhibiting abilities and due to its flavoring property used in food industry as a preservative (Churklam et al., 2020; Memar et al., 2017; Mousavi et al., 2020; Rezvi & Roy, 2019; Scaffaro et al., 2020; Sun et al., 2020).

**FIGURE 1:**
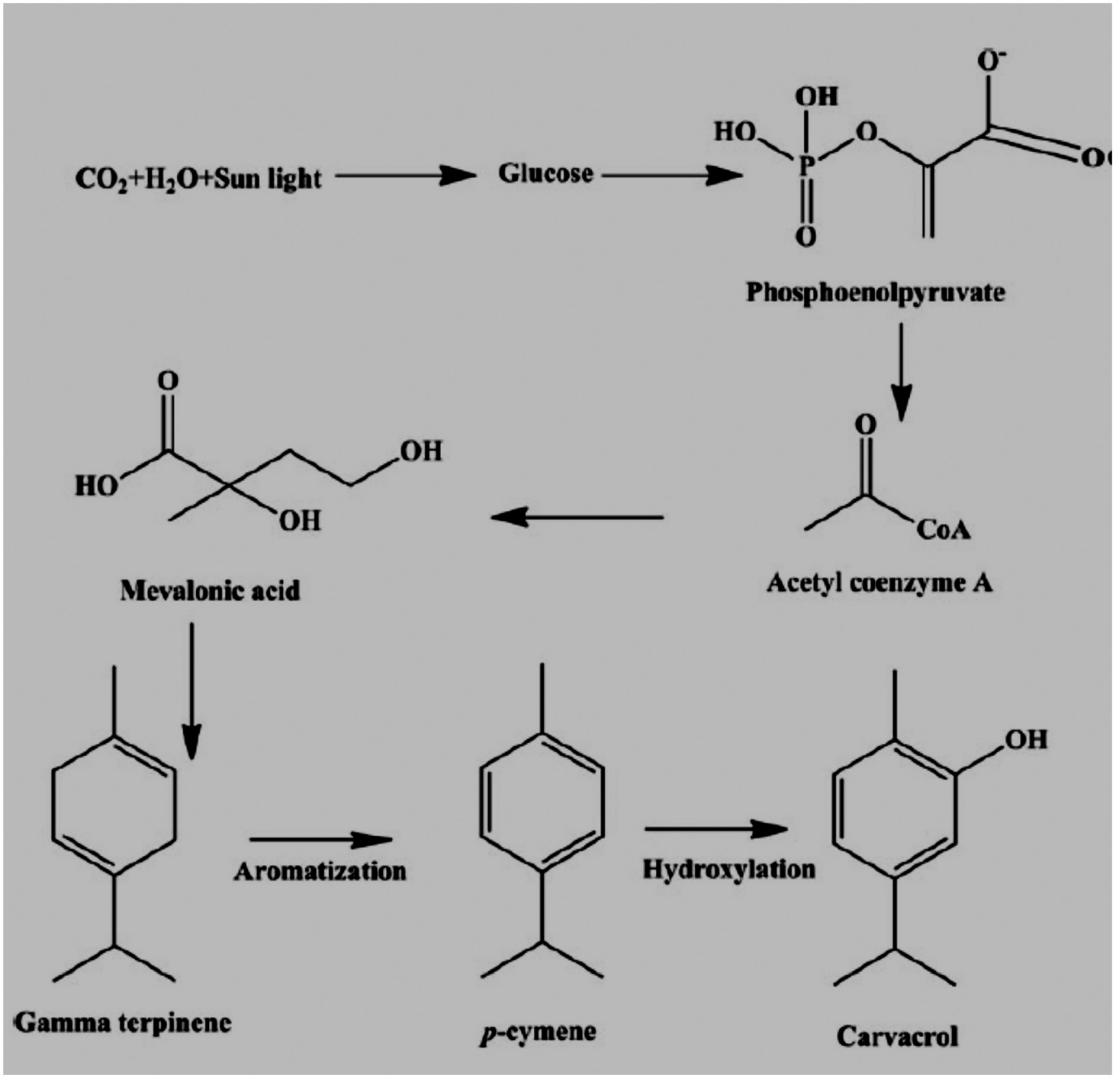
Carvacrol biosynthetic pathways via the mevalonate pathway.

Ezz‐Eldin et al. (2020) showed the antiproliferative, anti‐inflammatory, and pain‐relieving properties of carvacrol against bronchial asthma; bronchial asthma in animals was induced by an intranasal dose of ovalbumin. IN serum absolute eosinophil count (AEC) and immunoglobulin E (IgE) and inflammatory biomarkers like IL‐3, IL‐4, IL‐5, IL‐13, TNF‐α, IFN‐γ, and iNOS were determined in bronchoalveolar lavage fluid. The level of oxidative stress biomarkers was also measured. Results determined that carvacrol is a significant anti‐oxidant and antiproliferative agent (Ezz‐Eldin et al., 2020). Plants synthesized carvacrol via the mevalonate pathway. Glucose is first decarboxylated and acetylated to acetyl coenzyme A (acetyl CoA), which could then be turned into mevalonic acid. Aromatization converts mevalonic acid to gamma‐terpinene, which is then converted to p‐cymene. Carvacrol was produced by the hydroxylation of p‐cymene, as shown in Figure 1.

## 2 THERAPEUTIC PERSPECTIVES

### 2.1 Anti‐oxidant property

Oxidative stress is caused by the imbalance between oxygen reactive species and detoxification of the reactive intermediates via biological system ability; these free radicals damage different body cell molecules such as protein, lipids, and nucleic acids. Essential oils found in plants are natural anti‐oxidants that reduce cell damage caused by reactive species and prevent mutagenic and carcinogenic processes. Carvacrol has remarkably higher anti‐oxidative and hepatoprotective properties, which improves the activity of enzymatic anti‐oxidants (catalase, superoxide dismutase, and glutathione peroxidase) and the levels of nonenzymatic anti‐oxidants (vitamin C, reduces glutathione and vitamin E) (Gursul et al., 2019; Tohidi et al., 2020). *Origanum ones* L. contains the highest amount of carvacrol and showed significant anti‐oxidant and anticarcinogenic activities against the triple‐negative breast cancer MDA‐MB‐231 cell line than the human glioblastoma U87 cell line (Alagawany et al., 2015; Baranauskaite et al., 2017; Sharifi‐Rad et al., 2018).

The presence of hydroxyl group (OH) in carvacrol is the major reason for its radical scavenging activity (superoxide radicals, nitric oxide, and hydrogen peroxide). Its weak acid character facilitates hydrogen atoms donation to unpaired electrons, making another radical stabilized by electron scattering produced at molecule resonance structure (Cocolas et al., 2019; Mir et al., 2020). Carvacrol protects against oxidative stress made by restraint stress damages the brain, liver, and kidney. In the liver, acute pancreatitis leads to multiple organ dysfunction, and carvacrol has been investigated to have anti‐oxidative and hepatoprotective properties (Guarda et al., 2011; Mir et al., 2020; Rezvi & Roy, 2019; Samarghandian, Azimi‐Nezhad et al., 2016; Samarghandian, Farkhondeh et al., 2016).

A study conducted by Bakır et al. in 2016 analyzed the hepato‐protective effects of carvacrol on acute pancreatitis produced by cerulein and also explored the underlying mechanism. The rats were randomly divided into two groups (1) with no therapy, (2) provided with 50 μg/kg cerulean, (3) (50, 100, and 200 mg/kg) carvacrol, and (4) cerulein + carvacrol. Carvacrol decreased pancreatitis‐induced MDA and 8‐OH‐dG levels, and the activities of the liver SOD, CAT, and GSH‐Px increased. Carvacrol decreased the level of aspartate aminotransferase (AST), alanine aminotransferase (ALT), and lactic acid dehydrogenase (LDH) and improved the status of inflammation, necrosis, and coagulation in the liver (Bakır et al., 2016).

Carvacrol has anti‐inflammatory characteristics and protects against digestive and oral disease by acting as an agonist of the TRPA1 receptor (transient receptor potential cation channel, subfamily A, member 1). In 2016, Alvarenga et al. found that carvacrol had anti‐inflammatory property and helps us to maintain intestinal oral disease caused by irinotecan hydrochloride (CPT‐11) 75 mg/kg, i.p. for 4 days via TRPA1 activation. Carvacrol activated TRPA1, decreased some inflammatory indicators such as MPO, NF‐B, and COX‐2 receptors; reduced the release or production of pro‐inflammatory cytokines TNF‐, IL‐1, and KC, and lowered oxygen reactive species such as GSH, MDA, and NOx levels. Carvacrol also improved the blood bacterial count, leukogram, body mass variability, and survival rate, while also restoring villi structure in the small bowel (Alvarenga et al., 2016).

Another study conducted by Banik et al. in 2019 examined carvacrol's effects on apoptosis in PC12 cells induced by cadmium for 48 h in animals which increased the level of glutathione and glutathione reductase expression. Carvacrol also reduced the DNA fragmentation magnitude and also downregulated the level of mammalian target of rapamycin, protein kinase B (Akt), extracellular signal‐regulated kinase‐1, nuclear factor erythroid 2‐related factor 2 (Nrf2) expressions, and nuclear factor kappa‐light‐chain‐enhancer of activated B cells (NFКB). Carvacrol also reversed the action of cadmium via decreasing cytochrome c levels and also decreased the cleavage of caspase 3, an apoptosis‐inducing factor. Carvacrol increased the intracellular metallothionein content (Banik et al., 2019). The free hydroxyl functional group, specifically the molecular configuration, is the major contributing factor to its excellent antimicrobial capacity (Wang & Wu, 2021).

**FIGURE 2:**
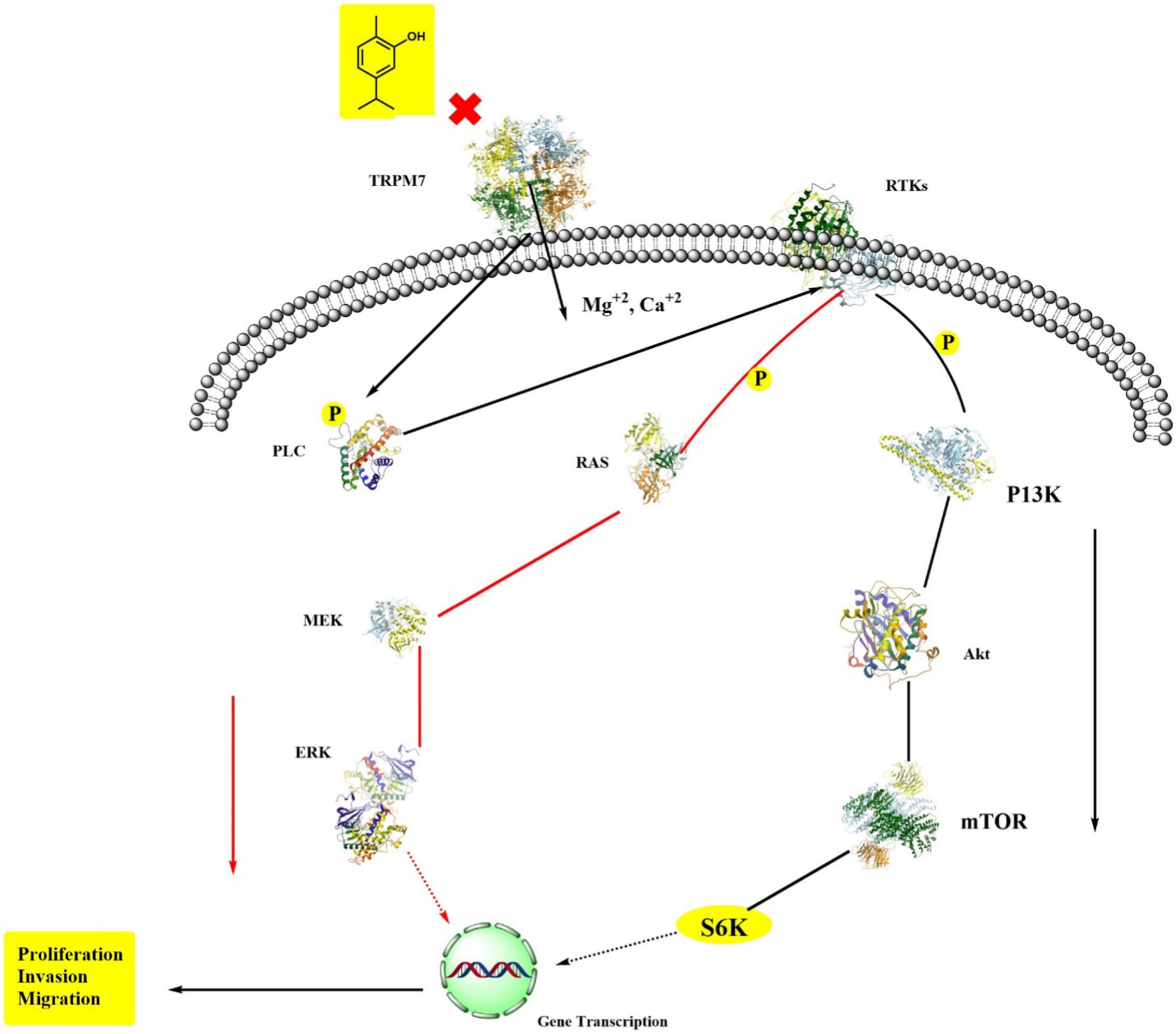
Anti‐oxidant potential of carvacrol.

**FIGURE 3:**
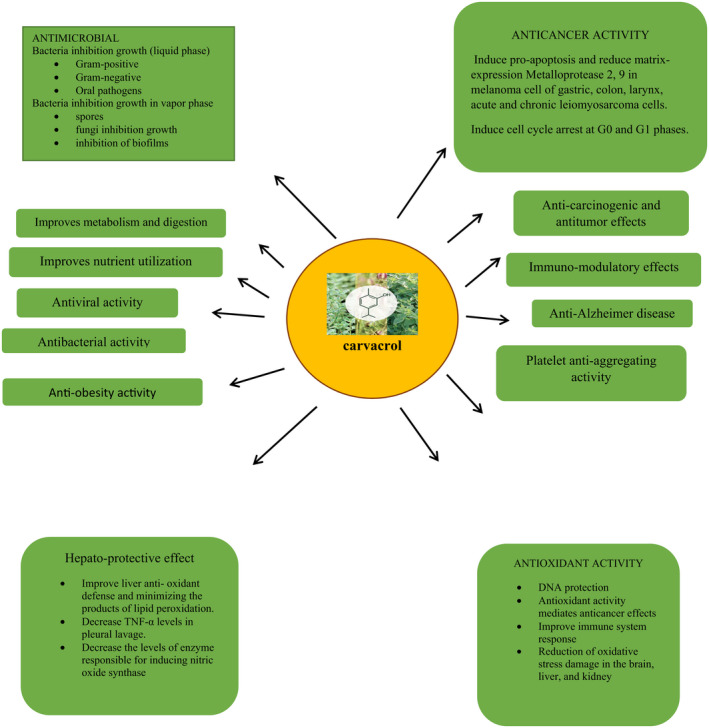
Health perspectives of carvacrol.

That is investigated that carvacrol increased the actions of anti‐oxidant enzymes and decreased lipid peroxidation levels in the liver. Carvacrol inhibits the liver damage caused by aging as it reduced oxidative stress in animal models (Samarghandian, Azimi‐Nezhad et al., 2016; Samarghandian, Farkhondeh et al., 2016). Arigesavan and Sudhandiran (2015) showed the anti‐oxidant property of carvacrol extracted from *O. vulgare* sp. and assessed its effect on colitis‐associated colon cancer (CACC) in male Fischer 344 rats. Carvacrol (50 mg) before and after tumor induction increased anti‐oxidant enzymes such as catalase, superoxide dismutase, and glutathione levels, reduced (myeloperoxidase, lipid peroxides, and nitric oxide), and restored the histological lesions in the colitis. Carvacrol also suppressed inducible nitric oxide synthase (iNOS) and pro‐inflammatory mediators such as interleukin‐1 beta (IL‐1β) (Arigesavan & Sudhandiran, 2015; Figures 2 and 3).

### 2.2 Anticancer activity

Carvacrol has anticancer ability against malignant cells via decreasing the expressions of matrix metalloprotease 2 and 9 (Bayir et al., 2019; Fan et al., 2015; Rezvi & Roy, 2019). Its antiproliferative activities induce apoptosis, which further increases the expression of pro‐apoptotic proteins. In cancer cells lines JAR and JEG3 cells, carvacrol induces calcium ions burden in the mitochondrial matrix via disrupting the mitochondrial membrane, suppresses extracellular signal‐regulated kinase 1/2 mitogen‐activated protein kinase (MAPK) signal transduction, also decreases the phosphoinositide 3‐kinase/protein kinase B, and increases phosphor‐P38 and c‐Jun N‐terminal kinase MAPK expressions (Chraibi et al., 2020; Lim et al., 2019). Carvacrol in normal cells (L92) was also found to induce apoptosis via mitochondrial membrane potential disruption, ROS generation, activation of caspase3, and DNA damage (Jamali et al., 2020). A study conducted by Fan et al. explored that carvacrol works as an anticancer agent in different human colon cell lines such as LoVo and HCT116 via decreasing the matrix metalloprotease 2 and 9, cell‐proliferation, cyclin B1 expression, and In causing In cell In cycle arrest at G2 and M phases. Additionally, it also increased phosphorylation of the extracellular‐regulated protein kinase B and downregulated Bcl‐2 expression (Fan et al., 2015). It also exhibited the dose‐dependent inhibition in tumor growth cells in prostate cancer cells (PC3 cells) (Trindade et al., 2019). In a study, carvacrol‐encapsulated nanoemulsion (CEN) formulated by a combination of polysorbate 80, lecithin, and MCT in lung A549 cells line in a dose‐dependent manner reduced the activation of MAPK, p38, and ERK and decreased the expression of CD31 and VEGF (Carvalho et al., 2020; Khan, Bhardwaj, Shukla, Lee, et al., 2019; Khan, Bhardwaj, Shukla, Min, et al., 2019; Khan, Singh et al., 2019). Carvacrol can inhibit prostate cancer progression by inducing programmed cell death and cell cycle arrest at G0 and M‐phases. In a dose‐ and time‐dependent manner, carvacrol exhibited protective effects against prostate cancer cells via lowering cell viability, increasing the rate of reactive oxygen species, and disrupting the mitochondrial membrane potential. Carvacrol induced cell cycle arrest at G0/G1 that declined increased CDK inhibitor p21 expression and decreased cyclin‐dependent kinase 4 (CDK4), and cyclin D1 expressions. Moreover, carvacrol inhibited Notch signaling in PC‐3 cells via downregulating Jagged‐1 and Notch‐1 (Karam et al., 2019).

Heidarian examined the dose‐dependent effects of carvacrol in human prostate cancer cell lines, which significantly reduced IL‐6 gene expression as compared to the control group in which IL‐6 protein reduced 41.5% and 52.7% at 360 and 420 μM. Carvacrol reduced cellular signaling proteins and gene expression and cellular signaling proteins. Further, it also caused a reduction in the cell survival rate, invasion, and proliferation rate (Bayir et al., 2019; Heidarian & Keloushadi, 2019). A study reported by Pakdemirli et al. in 2020 examined carvacrol effects on both HT‐29 and HCT‐116 via lowering the survival rate and proliferation rate (Pakdemirli et al., 2020). In different in vitro and in vivo studies of MDA‐MB 231 cells, carvacrol‐induced apoptosis lowered the mitochondrial membrane potential resulting in the release of cytochrome c from mitochondria, cleavage of PARP, and caspase activation (Arunasree, 2010). Certain available evidence showed that carvacrol has cell cycle G2 arresting ability against hepatic cancer cells via enhancing cell apoptosis, activation of the caspase‐3, cleavage of PARP, and decreasing gene expression of Bcl‐2. Carvacrol dose‐dependently lowers ERK1/2posphorylation, activates phosphorylation of p38, and alters the phosphorylation of the MAPK (Elshafie et al., 2017; Suntres et al., 2015; Yin et al., 2012). Same findings were discovered by Khan, Bahuguna et al. (2017), Khan, Khan, Ahmad et al. (2017) and Khan, Khan, Farooqui et al. (2017), who investigated that carvacrol in human prostate cancer cell lines induced apoptosis and exhibited cell cycle arrest at G0 and G1phases (Khan, Khan, Farooqui et al., 2017). Similarly, Khan, Bahuguna et al. (2017) found the dose‐ and time‐dependent effects of carvacrol on DU145 cells. Carvacrol induced apoptosis by nuclear condensation, caspase‐3 activation, and Annexin V‐FITC/PI‐positive cells. It disrupts the mitochondrial membrane potential and caused cell cycle arrest at G0 and G1 (Khan, Bahuguna et al., 2017).

Carvacrol has significant protective effects in reducing the side effects of chemotherapeutics such as irinotecan hydrochloride anticancer drugs that cause induction of intestinal mucositis. Irinotecan hydrochloride triggers inflammation and leads to cell‐damaging by the transient receptor potential cation channel, subfamily A, and member 1 receptor. Carvacrol reduced inflammatory biomarkers, such as nuclear factor κB and cyclooxygenase‐2, and levels of Nitric oxides, malondialdehyde, and glutathione create oxidative stress. It also acts as an agonist of the transient receptor potential cation channel (Alvarenga et al., 2016). In human cervical cancer HeLa cells, Potočnjak studied the anticancer role of carvacrol against human cervical cancer HeLa cells via decreasing the cell viability, inducing apoptosis, and inhibiting the mitogen‐activated protein kinase (Potočnjak et al., 2018; Zeytun & Özkorkmaz, 2021).

Carvacrol was found to have antitumor, antiproliferative, and apoptotic activity against human colon cancer cell lines LoVo and HCT116 when combined with thyme (Fan et al., 2015). Carvacrol decreased cancer cells proliferation and apoptosis via decreasing matrix metalloprotease (MMP‐2, MMP‐9) expressions while downregulating the Bcl‐2 expression and inducing phosphorylation of extracellular regulated protein kinase and protein kinase B(p‐Akt) at the molecular level (Fan et al., 2015).

**FIGURE 4:**
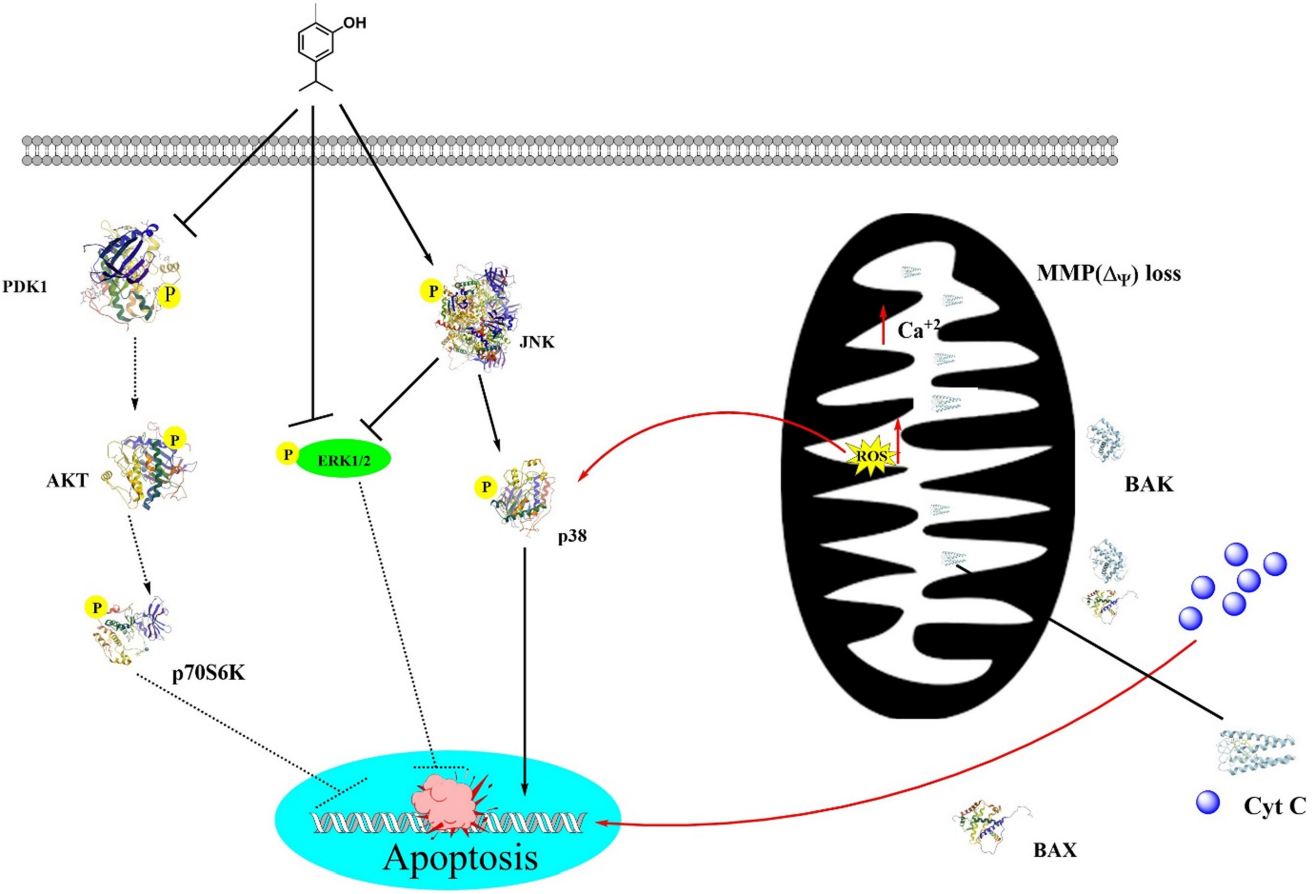
Scheme of anticancer‐induced reactions by carvacrol (modified from Liu et al., 2018).

Figure 4 depicts a model of the anticancer mechanism of carvacrol.

### 2.3 Antidiabetic role

Diabetic cardiomyopathy is characterized by an abnormality in diastolic relaxation and later heart failure without hypertension, dyslipidemia, and coronary artery disease. Hyperglycemia, insulin resistance, and increased insulin levels in the case of diabetes lead to the development of diabetic cardiomyopathy (Jia et al., 2018; Liu et al., 2020). Carvacrol restored PI3K/AKT signaling‐mediated GLUT4 membrane translocation and modulation of the PI3K. A study investigated the antidiabetic effects of intraperitoneal administration of carvacrol on streptozotocin‐generated type‐1 diabetes mellitus and type‐2 diabetes mellitus in db/db mouse model for 6 weeks. Carvacrol significantly improved blood glucose levels, cardiac fibrosis and reversed cardiac hypertrophy, Myh7, and Nppa mRNA expressions. Carvacrol improved levels of phosphorylated PI3K and PDK1 and decreased PTEN phosphorylation (Hou et al., 2019a, 2019b).

Combined antidiabetic and antihyperlipidemic effects of carvacrol 20 mg/kg per body weight with a thiazolidinedione and rosiglitazone 4 mg/kg per body weight fed to C57BL/6J mice. A high‐fat diet increased the plasma glucose level, decreased hemoglobin level, improved insulin levels, improved levels of glucose‐6‐phosphate and fructose‐1,6‐bisphosphatase involved in metabolism, and decreased glucose‐6‐phosphate dehydrogenase. Carvacrol also decreased the concentrations of alanine aminotransferase, alkaline phosphatase, aspartate aminotransferase, and gamma‐glutamyl transpeptidase. Carvacrol in combination with a thiazolidinedione or rosiglitazone restored liver function, insulin level, and plasma glucose level (Ezhumalai et al., 2014). Similarly, different doses of thymoquinone (25 and 50 mg/kg BW) were treated with experimental subjects and showed momentous reductions in serum glucose level, cholesterol, low‐density lipoprotein, liver enzymes, and enhancement in insulin level (Bayramoglu et al., 2014). In a recent study, Shoorei et al. (2019) studied the antidiabetic effects of carvacrol in 32 adult male Wistar diabetic rats (50 mg/kg BW streptozotocin). Carvacrol enhanced the activities of glutathione peroxidase enzymes and superoxide dismutase, lowered tissue malondialdehyde, rate of germ cells apoptosis, and Bax expressions, and improved Bcl‐2 protein expression (Shoorei et al., 2019). In 12‐week‐old spontaneously hypertensive rats (SHR), Wistar Kyoto (WKY) rats, administrated thymoquinone (50 or 100 mg/kg/day) lowered blood pressure, improved the intracavernosal pressure, enhanced the acetylcholine‐ and SNP‐induced relaxation responses, and improved the erectile functions, along with causing a reduction in endothelial, dysfunction, smooth muscle cell hypercontractility, and superoxide anion generation (Gonçalves et al., 2019). Amiri and Akbari in 2018 investigated the antidiabetic effects of carvacrol (25 and 50 mg/kg BW) given to diabetic rats induced by streptozotocin for 7 days. Carvacrol lowered the level of glucose, serum total cholesterol, alanine aminotransferase, aspartate aminotransferase, lactate dehydrogenase, and body weight (Amiri & Akbari, 2018; Deng et al., 2013).

### 2.4 Anti‐obesity effects

De novo lipogenesis is the process of formation of new adipose cells derived from the ChREBP transcription factor. In this process, white adipocytes are engaged by one large fat droplet. Spalletta et al. (2018) investigated that carvacrol inhibited fat accumulation in humans 30% in Wharton' jelly‐derived mesenchymal stem cells and 40% in murine 3T3‐L1 cells. In addition, it also reduced autophagy and ChREBP expression (Spalletta et al., 2018).

Carvacrol can control obesity by inhibiting intracellular fat accumulation and adipocyte differentiation as evidenced in high‐fat‐diet‐induced male C57BL/6N mice embryo 3T3‐L1 cells and the mechanism involved in gene expression in thermogenesis, adipogenesis, and inflammation. Carvacrol inhibited visceral adipogenesis through suppression of bone morphogenic protein‐, galanin‐mediated signaling, and fibroblast growth factor‐1. Carvacrol inhibited toll‐like receptor (TLR2 and TLR4)‐mediated signaling and improved pro‐inflammatory cytokines formation in visceral adipose tissues (Cho et al., 2012). Carvacrol in combination with rosiglitazone on diabetic mice C57BL/6J showed a reduction in triglycerides, low‐density lipoproteins cholesterol, total cholesterol, phospholipids, and free fatty acids (Ezhumalai et al., 2015). A study conducted by Umaya and Manpal found that carvacrol has an anti‐obesity role on embryo 3T3‐L1 cells via lowering fat deposition in cells and visceral fats and also improving free fatty acids, liver cholesterol, and HDL‐cholesterol. Carvacrol reduced adipogenesis‐related gene fibroblast growth factor receptor in visceral adipose tissues and galanin receptor 1 and 2 expressions (Suganthi & Manpal, 2013). In a high‐fat‐induced C57BL/6J mice study, carvacrol (20 mg/kg BW) used in combination with thiazolidinediones and rosiglitazone lowered the plasma glucose level, increased hemoglobin level, and increased as well as also alanine aminotransferase, alkaline phosphatase, and aspartate aminotransferase and gamma‐glutamyl transpeptidase (Ezhumalai et al., 2014). In addition, carvacrol (25 and 50 mg/kg) was supplemented to streptozotocin‐induced diabetes rats for 7 days and found that carvacrol significantly reduced the level of glucose, serum total cholesterol, and body weight changes (Amiri & Akbari, 2018).

### 2.5 Pain management

A study conducted by Guimarães and fellows showed the carvacrol interaction with IL‐10 and GABAA in which carvacrol 12.5–50 mg/kg once daily for 15 days in mice that significantly decreased mechanical hyperalgesia, improved use paw, spontaneous and palpation‐induced nociception. Carvacrol also decreased neurons in the lumbar spinal cord, nucleus raphe Magnus and locus coeruleus, as well as activated peri‐aqueductal gray (Guimarães et al., 2014). Guimarães et al. (2014) investigated that carvacrol has been used to cure pain caused by inflammation and mechanical hyper‐nociception. Carvacrol is also associated with reducing pains in intraoral and oro‐facial structures such as the mouth, face, head, and neck (Silva et al., 2016). Likewise, administrated carvacrol at the rate of 100 mg/kg exhibited antinociceptive effects via lowering the inflammatory mediators and pain (Milovanović et al., 2016). In another study conducted by Klein et al. (2013), they examined analgesic and anti‐inflammatory effects of carvacrol along with eugenol worked as an agonist of transient receptor potential cation channel, subfamily V, member 3 (TRPV3s), which is involved in heat and pain transduction showed that heat hyperalgesia involved TRPV3‐mediated development of thermal gating of TRPV1 expressed in lingual polymodal nociceptors (Klein et al., 2013).

### 2.6 Anti‐inflammatory effects

In asthma, airway inflammation can be suppressed by peroxisome proliferator‐activated receptor‐alpha (PPAR‐α) agonists as it reduces the release of inflammatory mediators majorly involved in asthma (Gholijani et al., 2016; Rolim et al., 2019; Sun et al., 2020). Carvacrol protects from intestinal mucositis as it acts as an agonist of transient receptor potential cation channel, subfamily A, member 1(TRPA1). Alvarenga et al. (2016) determined anti‐inflammatory actions via activating the TRPA1 in intestinal mucositis induced by irinotecan hydrochloride (CPT‐11) at 75 mg/kg. It lowered MPO, NF‐ κB, C‐2 receptor, and production of pro‐inflammatory cytokines and lowered the malondialdehyde and nitric oxide level. It also restored villi architecture in the small intestine and side by side improved the blood bacterial count, leukogram, body mass variation, and survival rate. Carvacrol also significantly reduced nitric oxide, lipid peroxides, interleukin‐1 beta, and myeloperoxidase (Alvarenga et al., 2016; Arigesavan & Sudhandiran, 2015). Carvacrol also significantly reduced nitric oxide, lipid peroxides, interleukin‐1 beta, and myeloperoxidase (Arigesavan & Sudhandiran, 2015). Somensi et al. (2019) investigated the molecular pathways of carvacrol action on LPS‐mediated pro‐inflammatory activation of RAW 264.7 macrophages. Preincubation with 100 μM carvacrol prevented ERK1/2 phosphorylation and inhibited NF‐kB (p65) translocation from cytoplasm to nucleus, but it showed no response on p38 and JNK activation (Somensi et al., 2019).

Banik et al. (2019) explored the anti‐inflammatory and antitoxic effects of carvacrol and that it reduced oxidative damage in PC12 cells due to cadmium against caspase‐dependent and apoptosis‐independent pathways. Carvacrol exposure increased glutathione reductase expressions and cellular level of glutathione as well as ameliorated DNA fragmentation magnitude caused by cadmium. Carvacrol improved nuclear factor kappa‐light‐chain‐enhancer of activated B cells (NFКB), downregulation of mammalian target of rapamycin (mTOR), protein kinase B (Akt), and extracellular signal‐regulated kinase‐1 (ERK‐1). Carvacrol also suppressed the cleavage of caspase 3, reduced the apoptosis‐inducing factor (AIF) and cytosolic levels of cytochrome c, and increased the intracellular metallothionein content (Banik et al., 2019). A group of researchers and investigators determined the protective effects of carvacrol against cisplatin resistance in HeLa cells via ERK1/2‐dependent autophagy. Both compounds increased cisplatin‐induced light chain 3 beta expressions enhanced by MEK inhibition (Potočnjak et al., 2018).

Therapeutic effects of carvacrol (75 mg/kg) were studied by Zeytun and Özkorkmaz in 2021 against inflammation of Wistar albino rats and induced positive caspase 3 expression within apoptotic cells of the epithelial layer and connective tissues. Carvacrol increased mitotic activities and degenerative changes. Vascular endothelial growth factor was also seen in the papillary region of epithelium and also dilated vascular endothelial cells (Ezz‐Eldin et al., 2020). Rheumatoid arthritis (RA) is the inflammation of the joints and carvacrol has been proved by different studies that it can reduce inflammation in fibroblast‐like synoviocytes. A study conducted by Li et al. in 2019 showed that carvacrol inhibited LPS‐induced inflammation, cell proliferation, and migration in rheumatoid arthritis‐induced fibroblast‐like synoviocytes. Carvacrol decreased the production of inflammatory cytokines, as well as matrix metalloprotease such as MMP‐1, MMP‐3, and MMP‐13. Moreover, it also inhibited the activation pathways of TLR4/MyD88/NF‐κB, p38, and ERK1/2, respectively (Li et al., 2019). Encapsulated carvacrol in bovine serum albumin (BSA) is used to examine its therapeutic and immunomodulatory effects in adjuvant‐induced arthritis (AIA) in Sprague Dawley rats. Carvacrol‐loaded BSA nanoparticles significantly decreased clinical severity score, erythrocyte sedimentation rate, nitric oxide production, and interleukin (IL)‐17 gene expression (Gholijani et al., 2020).

Carvacrol modulates the neuro‐transmitter pathways, such as serotoninergic, dopaminergic, and γ‐amino butyric acid use in the release and production of inflammatory mediators (Bayir et al., 2019; Lima et al., 2017; Rezvi & Roy, 2019; Sharifi‐Rad et al., 2018).

### 2.7 Antimicrobial activity

Carvacrol has strong antibacterial activity against *Staphylococcus epidermis*, *Staphylococcus aureus*, *Klebsiella pneumonia*, *Escherichia coli*, *Streptococcus pneumonia*, *Proteus mirabilis*, Serratia spp., Enterobacter spp. (Alagawany et al., 2015). Carvacrol antimicrobial actions showed by Rodriguez‐Garcia et al. in 2016 with thymol on *S. aureus*, *Clostridium perfringens*, and *Pseudomonas aeruginosa* (Rodriguez‐Garcia et al., 2016). Carvacrol also has antifungal actions on different microbes including *Aspergillus niger*, *Aspergillus flavus*, Candida spp., *Alternaria alternata*, *Trichoderma viride*, *Penicillium rubrum*, and dermatophytes. Carvacrol antimicrobial activity extended to pathogens of fungal plants including *Colletotrichum acutatum*, *Colletotrichum fragariae*, and *Colletotrichum gloeosporioides* (Alagawany et al., 2015; Rodriguez‐Garcia et al., 2016).

Carvacrol and thymol exhibited antimicrobial activity against *Salmonella enterica* or *S. aureus*. Carvacrol, thymol, and thymol/carvacrol liposome minimum inhibitory concentration of *S. aureus* was 0.662 mg/ml and for salmonella pool 0.331 mg/ml. 1 min and 10 min treatment of carvacrol at a concentration of 2.0 against *S. aureus*. It is concluded that contact times and antimicrobial concentrations of carvacrol, thymol, and TCL can be used in food products to avoid biofilm formation at the initial stage of microbial attachment (Engel et al., 2017). The encapsulated form of carvacrol in poly(DL‐lactide‐co‐glycolide) (PLDA) nanocapsules is widely used as it has a suitable drug delivery system that interacts with the biofilm formation associated with different infections with biofilm viscoelasticity modifier (Iannitelli et al., 2011). Carvacrol downregulates the genes that are involved in aerobic metabolism, and majorly target the cytoplasmic membrane of *E. coli* (Siroli et al., 2018). In a study reported by Sun et al., microencapsulated carvacrol exhibited antimicrobial activity against (62.0%), *Salmonella typhi* (68.0%), *Listeria monocytogenes* (89.0%), and *S. aureus* (49.0%) (Fonseca et al., 2019). The synergic effects of carvacrol with thymol have the higher antibacterial and efflux pump inhibiting capacities in the case of foodborne pathogens (Miladi et al., 2016). Beaubrun et al. (2018) showed that carvacrol exhibited antimicrobial activity against *S. enterica* pre‐enriched with or without 2% (v/v) corn oil.

The synergistic effect of sodium chloride and essential oil containing carvacrol and thymol showed antimicrobial action on *E. coli O157:H7*, *L. monocytogenes*, and *S. aureus*. A combined form of carvacrol/thymol (2.0 mM) with NaCl (≥3%) induced membrane disruption, and bacterial cells lost their ability to maintain osmotic balance and became completely inactivated (Kim et al., 2017; Mazarei & Rafati, 2019). Carvacrol combination with hydroxypropyl methylcellulose, tapioca starch, glycerol, and potassium sorbate showed significant microbial effects for *Zygosaccharomyces bailii*, *Pseudomonas fluorescens*, and *Lactobacillus plantarum* as compared to films only containing KS., *L. plantarum*, and *P. fluorescens* in comparison with films containing only KS (Alzate et al., 2017).

Carvacrol also showed antimicrobial potential on *S. aureus*, whereas carvacrol has also higher antivirulence and antibiofilm actions against uropathogenic *E. coli* (Lee et al., 2017; Monzote et al., 2018). Carvacrol also inhibits virus growth such as human rotavirus (RV), especially of acyclovir‐resistant herpes simplex virus type 1 (ACVRHHV‐1), human respiratory syncytial virus (HRSV), and pandemic H1N1 virus documented by different scientific researches (Gilling et al., 2014; Pilau et al., 2011; Vimalanathan & Hudson, 2012). In another piece of evidence, Wei et al. (2017) supplemented animal feed with 100 mg/kg of carvacrol and thymol in (1:1) for 14 days reduced intestinal oxidative stress and inflammation in piglets, which significantly reduced TNF α, level of IL1 β, IL‐6 via decreasing mRNA levels as piglets suffer intestinal dysfunctioning that effects and compromises their performance (Wei et al., 2017).

### 2.8 Neuroprotective role

The neuroprotective role of carvacrol was examined by Guan et al. in 2019 against ischemic stroke, leading to hippocampal neuron damage and impairment in gerbils brain tissue improved. Carvacrol decreased levels of lipid peroxide, reduced cell death, and increased the expression of glutathione peroxidase 4 inhibited by ferroptosis (Guan et al., 2019). In recent findings, Raeini et al. (2019) evaluated that carvacrol has a significant ability to reduce neuronal necrosis and malondialdehyde (MDA) and elevated the levels of superoxide dismutase and catalase activities in the hippocampus of 48 male Wistar rats. Carvacrol improved cognitive functions, spatial learning, and memory capacities (Raeini et al., 2019). Carvacrol deters transient receptor potential melastatin 7 (TRPM7), which involved in the homeostasis of calcium and zinc. A study investigated the neuroprotective effects of carvacrol 50 mg/kg on cerebral ischemia through blockade of zinc influx on 8‐week‐old male Sprague–Dawley rats. Furthermore, carvacrol decreased oxidative damage, microglial activation, number of degenerating neurons, and zinc translocation through downregulating TRPM7 channels (Hong et al., 2018).

Zotti et al. (2013) documented that the administration of 12.5 mg/kg of carvacrol has an impact on neurochemistry and behavioral outcome in the prefrontal cortex and hippocampus of animal models, which increased serotonin and dopamine levels. Likewise, 450 mg/kg of carvacrol significantly reduced dopamine in the hippocampus of animals. Results suggested that it is a potent brain‐activating molecule that modulates neurotransmitters and neuron activities (Zotti et al., 2013). The peripheral neuro‐degenerative process is considered important for regenerating peripheral nerves genetically or mechanically. Carvacrol regulates transient receptor potential ankyrin 1 (TRPA1), TRP melastatin M7 (TRPM7), TRP canonical 1 (TRPC1), and TRP vanilloid 3 (TRPV3). A study investigated the regulatory effect of carvacrol on TRPM7‐dependent neurodegenerative process carvacrol specificity Schwann cells. Carvacrol significantly suppressed the morphometric indices, such as myelin, stripe, ovoid, and neurofilament indices. Moreover, carvacrol inhibited TRPM7 upregulation in Schwann cells and protected against the peripheral neurodegenerative process (Chun et al., 2019).

Carvacrol also protects from 6‐hydroxydopamine‐induced neurotoxicity as evidenced by various studies on Parkinson's disease; Manouchehrabadi et al. (2019) investigated the neuroprotective effects of carvacrol on Parkinson's disease models in in vitro and in vivo experiments. In in vitro experiment, post‐treatment carvacrol protects the adrenal pheochromocytoma PC12 cells of animals from 6‐hydroxydopamine‐induced neurotoxicity by toxicity induced by a reduction in intracellular reactive oxygen species, increasing cell viability and reduced intra‐cellular lipid In peroxidation In and In annexin In positive In cells In. Additionally, carvacrol protects against neurodegenerative diseases as it improved catalepsy, akinesia, bradykinesia, locomotor activity, and motor coordination. It also reduced the apo‐morphine, decreased the level of malondialdehyde, and increased the level of reduced glutathione content (Manouchehrabadi et al., 2019).

### 2.9 Hepatoprotective and gastroprotective role

Carvacrol protects from hepatotoxicity caused by D‐galactosamine (D‐GalN). It suppresses the CYP2E1 and upregulates PPAR‐α, mRNA, and protein expressions (Alagawany et al., 2015; Aristatile et al., 2014; Jamali et al., 2020). Carvacrol also reduces restraint stress induced by chronic exposure to oxidative stress leading to tissue damage in the brain, liver, and kidney. A study indicated that carvacrol prevents oxidative damage and restraint stress. Carvacrol was administered systemically for 21 days in animals and the levels of malondialdehyde (MDA) decreased glutathione, SOD, restraint stress, glutathione peroxidase, and glutathione reductase measured in the brain, liver, and kidney of animals. Results showed that in stressed animals' MDA, the level was higher and the levels of GSH and anti‐oxidant enzymes were significantly lower (Samarghandian, Azimi‐Nezhad et al., 2016; Samarghandian, Farkhondeh et al., 2016).

Another study investigated 15 mg/kg of carvacrol's protective effects on age‐associated changes in the action of anti‐oxidant enzymes and levels of lipid per‐oxidation at different ages (2, 10, 20 months) of rats. Results showed more improvement in the actions of anti‐oxidant enzymes of 20 months old rats and carvacrol helped in decreasing lipid peroxidation of 10 and 20 months old rats (Samarghandian, Azimi‐Nezhad et al., 2016). Carvacrol effects on acute pancreatitis (AP) induced by cerulean in animal models analyzed by Bakir et al. in 2016 showed the dose‐dependent manner of carvacrol decreased pancreatitis‐induced malondialdehyde and 8‐hydroxydeoxyguanosine levels as it improved the levels of anti‐oxidant enzymes and decreased the levels of AST, ALT, and LDH (Bakır et al., 2016). Carvacrol also treats cisplatin‐induced acute kidney injury via suppression of ERK and activating PI3K/Akt. Renal oxidative stress increased the expression of 4‐hydroxynonenal, 3‐nitrotyrosine, cytochrome P450 E1 (CYP2E1), heme‐oxygenase‐1 (HO‐1) and expressions of phosphorylated nuclear factor‐kappaB p65 and tumor necrosis factor‐α in animal. The results indicated that acute renal injury suppressed oxidative stress, apoptosis, and inflammation through the modulation of the ERK and PI3K/Akt pathways (Potočnjak & Domitrović, 2016).

A study conducted by Shanmugam et al. in 2019 indicated that carvacrol actions on liver tissues against toxicity was caused by sodium fluoride in rats. Carvacrol supplementation normalized all the anti‐oxidant enzymes and hepatic markers in NaF toxicity rats. Diphenyl‐1‐picrylhydrazyl (DPPH), hydrogen peroxide (H_2_O_2_), and hydroxyl radical activities showed potent free‐radical scavenging activities. The study concluded that carvacrol modulated the anti‐oxidant enzymes and hepatic stress markers in NaF rats (Shanmugam et al., 2019). Carvacrol also protects from gastric ulcers and has become a worldwide health problem. A peptic ulcer is one of the gastric problems caused by Helicobacter pylori, smoking, stress, alcohol, and nonsteroidal anti‐inflammatory drugs (NSAID). A study investigated the gastroprotective effects of carvacrol in rodents in which NSAID, ischemia, and reperfusion made gastric lesions. The results demonstrated that carvacrol promoted a marked gastroprotection mediated by endogenous prostaglandins, increase in mucus production, KATP channels opening, NO synthase activation, and anti‐oxidant properties (Oliveira et al., 2012).

Carvacrol has significant protective effects in reducing the side effects of chemotherapeutics such as irinotecan hydrochloride anticancer drugs that cause induction of intestinal mucositis. Irinotecan hydrochloride triggers inflammation and leads to cell damaging via the transient receptor potential cation channel, subfamily A, and member 1 receptor. Carvacrol reduced inflammatory biomarkers, such as nuclear factor κB and cyclooxygenase‐2, and levels of nitric oxides, malondialdehyde, and glutathione create oxidative stress. It also acts as an agonist of the transient receptor potential cation channel. Carvacrol also restored the tissue architecture of the villi and crypts in the small intestine and side by side improved the blood bacterial count, leukogram, body mass variation, and survival rate (Alvarenga et al., 2016). Arigesavan and Sudhandiran (2015) showed the anti‐inflammatory effects of carvacrol in the colon of Fischer 344 rats against inflammation induced by 1, 2‐dimethyl hydrazine plus dextran sodium sulfate. F344 rats were given three subcutaneous injections of DMH (40 mg/kg body wt) in the first week to F344 rats and free access to drinking water containing 1% DSS for the next 1 week was also given for 7–14 days as three cycles. 50 mg/kg body weight (o.p) carvacrol was administrated before and after tumor induction. Carvacrol‐treated groups suppress the inflammation in DMH/DSS‐induced animals, increased anti‐oxidant status; developed an endogenous anti‐oxidant system was observed and restorative histological lesions. Carvacrol also increased significantly the level of anti‐oxidant enzymes such as glutathione levels, superoxide dismutase, catalase, reduced nitric oxide, lipid peroxides, interleukin‐1 beta, and myeloperoxidase as compared to DMH/DSS induced rats (Arigesavan & Sudhandiran, 2015).

### 2.10 Cardioprotective

Carvacrol works as an agonist/antagonist of different voltage‐dependent calcium channels and TRP channels due to their involvement in bradycardia and peripheral vasodilation (Dantas et al., 2015). A study investigated the vasorelaxant effects of carvacrol along with thymol in the aorta and putative mechanism in rats as carvacrol and thymol 1000 μm abolished the phenylephrine‐created endothelium‐containing ring contraction in calcium‐free medium and at 400 μm, both reduced contraction induced by CaCl_2_ in Ca^2+^‐free medium, and also 1000 μm of carvacrol significantly reduced phorbol butyrate (1 μm)‐induced contraction, but effects could be seen on altering the resting potential of vascular smooth muscles cells (Peixoto‐Neves et al., 2010). Hotta et al. (2010) showed that carvacrol can suppress cyclooxygenase‐2 expression and activate peroxisome proliferator‐activated receptors PPARα and PPARγ (Hotta et al., 2010).

Protection of the heart function, enhancement in SOD and CAT levels, attenuation in myocardial infarct size, reduction in MDA level, and especially decreased cardiomyocytes apoptosis were reported after carvacrol treatment. Carvacrol showed the cardioprotective effects against myocardial ischemia injury in animals by activating Akt/eNOS and MAPK/ERK and upregulating the phosphorylated ERK (p‐ERK). It also showed no impact on onp38 mitogen‐activated protein kinase (p38MAPK) and c‐Jun N‐terminal kinase (JNK) (Chen et al., 2017). A study conducted by Yu et al. investigated the cardio‐protective effects of carvacrol against myocardial ischemia in animals via multiple mechanisms such as reduction in the cardiac infarct size, lactate dehydrogenase, and cardiac troponin T, enhancement in enzymatic anti‐oxidant concentrations, reduction in malondialdehyde, inhibition of expression of caspase‐3, Bax, and increase in the level of Bcl‐2 protein (Yu et al., 2013).

A study conducted by El‐Sayed et al. in 2016 assessed the protective effect of carvacrol in cardiotoxicity induced by doxorubicin (10 mg/kg). Supplementation of carvacrol (25 mg/kg p.o.) in combination with thymol (20 mg/kg p.o.) for 14 days lowered oxidative stress and improved the functioning of the heart (El‐Sayed et al., 2016), whereas Song et al. described that carvacrol in wild‐type male C57 BL/6 mice prevents myocardial ischemia–reperfusion injury via lowering myocardial infarct size, oxidative stress level, and cardiac myocyte apoptosis rate (Song et al., 2013). Lead is known to cause constriction of heart vessels, whereas Shabir found contracting response of aortal rings of rats induced by Pb(II) via elevation of ROS and depletion of nitric oxide. Carvacrol treatment prevented these contracting responses of aortal rings in rats (Shabir et al., 2014). Similarly, cyclophosphamide increased the serum alanine transaminase, aspartate transaminase, lactate dehydrogenase, creatine kinase‐MB, total oxidant state, and oxidative stress index in experimental animals, whereas different concentrations of carvacrol at 5.0 and 10 mg/kg reverted these changes (Cetik et al., 2015).

Left ventricular hypertrophy is caused by cardiomyocytes apoptosis induced by Bcl‐2 family members involved in regulating mitochondrial pathways of apoptosis. Carvacrol has a positive impact on the transcription level of antiapoptotic (Bcl2 and Bcl‐xL) members and pro‐apoptotic (Bad and Bax) of Bcl‐2 family in hypertrophied hearts of male Wistar rats as well as lowered heart weight to body weight ratio, and Bad mRNA level (Sadeghzadeh et al., 2018). Carvacrol inhibited platelet‐derived growth factor (PDGF)‐BB which stimulated aortic smooth muscle cell proliferation and migration dose‐dependently as well as decreased aortic sprout outgrowth and reduced the phosphorylation of MAPK and ERK1/2 (Lee et al., 2015).

A study conducted by Jamhiri and fellows found that different in vitro and in vivo administrations of carvacrol (25, 50, and 75 mg/kg/day) against left ventricular hypertrophy of male Wistar rats reduced blood pressure, heart rate, and heart weight to the body weight ratio. In the in vitro study, carvacrol momentously lowered the H9c2 cell size as compared to Ang II‐treated cells. Furthermore, 50 and 75 mg/kg/day doses significantly inhibited the atrial natriuretic peptide and lowered the number of apoptotic cells (Alves et al., 2016). Carvacrol administration with different doses (25, 50 and 75 mg/kg) exerts a cardio‐protective role against cardiac‐hypertrophy of male Wistar rats via increasing catalase and mRNA expression (Jamhiri et al., 2019; de Souza Polli et al., 2019; Shahrokhi Raeini et al., 2018).

Carvacrol has protective effects on myocardial ischemia–reperfusion in wild‐type C57 BL/6 mice via descending coronary artery occlusion, reducing the average area of mitochondria, the number of granules in the mitochondrial matrix, the number of perimitochondrial lipid droplets, and the percentage of mitochondrial fission (Wang et al., 2013).

## 3 IMMUNOMODULATORY ROLE

Carvacrol action can modulate immune responses via different inflammatory parameters such as proliferation of T‐cells, isolated polymorph nuclear neutrophils IL‐2 and TNF‐α cytokines production, and ROS generating from whole blood phagocytes. Ezz‐Eldin et al. (2020) investigated the immunomodulatory effect of carvacrol against bronchial asthma induced by an intranasal dose of ovalbumin. It significantly caused a reduction in absolute eosinophil count, absolute eosinophil count, immunoglobulin E, inflammatory biomarkers (TNF‐α, L‐4, IL‐5, IL‐13, and interferon‐gamma), and enhancement in anti‐oxidant enzymes further that prevent from the inflammatory symptoms in asthma (Ezz‐Eldin et al., 2020). Combination of carvacrol with essential oils (*Foeniculum vulgare*, *Saturea cuneifolia*, and *Origanum munitiflorum*) can inhibit ROS production, T‐cells proliferation, and pro‐inflammatory cytokines (Khazdair et al., 2018; Orhan et al., 2016). Encapsulated carvacrol (250–650 μg/g) in necrotic enteritis animal disease caused by *C. perfringens* in chicken intestine prevented the immune‐mediated responses (Liu et al., 2016). Thymol and carvacrol in human mesenchymal stromal cells protect from oxidative stress‐related damage and cytotoxicity and preserve cell morphology (Bouhtit et al., 2019).

Tolerogenic dendritic cells (DCs) are a condition that leads to the induction of dampened pathogenic T cell responses and FoxP3+ regulatory T cells. Spiering et al. (2012) investigated the immunomodulatory properties of carvacrol by suppressing autoimmune arthritis in a mouse model. Amirghofran et al. (2016) also investigated carvacrol and thymol actions on DCs maturation and T cell activation. Both compounds also inhibited the mitogenic and allogeneic T‐cells responses and release of cytokines (Amirghofran et al., 2016). Carvacrol was also found to lower the vascular cell adhesion molecule 1 (VCAM‐1), monocyte chemo‐attractant protein (MCP‐1), intracellular cell adhesion molecule 1 (ICAM‐1), interferon gamma‐induced protein 10, interferon‐inducible T‐cell alpha chemo‐attractant(I‐TAC), etc., as well as also decrease the remodeling biomarkers such as collagen I, III, epidermal growth factor receptor(EGFR), matrix metalloproteinase 1, and plasminogen activator inhibitor 1 (Han & Parker, 2017).

By investigating thymol and carvacrol (25 μg/ml) effects against Jurkat leukemia cells in in vitro models, Gholijani et al. in 2015 concluded that both compounds reduced IL‐2 and IFN‐γ production via downregulating AP‐1 and NFAT‐2 transcription factors (Gholijani et al.,  2015). A study reported by Gandhi et al. found that carvacrol also regulates cytokine production, inhibits ROS accumulations, and inactivates eosinophils migration lungs. EO suppressed cytokine production, pro‐inflammatory and anti‐inflammatory mediators formation, and accumulation (Gandhi et al., 2020).

Carvacrol also protects in case of multiple sclerosis (MS) development by modulating pro‐ and anti‐inflammatory properties (TGF‐β, IL‐4, and IL‐10). Mahmoodi et al. (2019) studied carvacrol 5 and 10 mg/kg dose effect on auto‐immune encephalomyelitis and exhibited immune‐modulating actions on pro‐ and anti‐inflammatory cytokines. Carvacrol also improved pathological problems and improved clinical symptoms in patients (Gholijani & Amirghofran, 2016; Mahmoodi et al., 2019). Similarly, carvacrol (73 mg/kg) in combination with pomegranate (225 mg/kg/day) was studied against methotrexate (MTX)‐induced intestinal damage in 32 male Sprague–Dawley rats, using immunohistochemical and histopathological techniques (Türkcü et al., 2015). Carvacrol effects on cytokines genes expression in splenocytes of asthmatic mice were studied by Kianmehr et al. (2016) in rats in which asthma is induced by ovalbumin (OVA) and it was concluded that carvacrol significantly modulated the immune response by decreasing IL‐4, IL‐17, and TGF‐β gene expressions and increased IFN‐γ and FOXP3 (Kianmehr et al., 2016).

Khazdair and Boskabady (2019a, 2019b) investigated carvacrol effects on serum levels of interferon‐gamma (IFNγ), interleukins (IL‐2, IL‐4, IL‐6, IL‐8, and IL‐10), and pulmonary function tests in 22 patients exposed to sulfur mustard (SM) 27–30 years in a double‐blind manner for 2 months divided into placebo and carvacrol 1.2 mg/kg/day. It was concluded that carvacrol reduced inflammatory cytokines, while increased anti‐inflammatory cytokines and improved PFT tests in SM‐induced lung injury (Khazdair & Boskabady, 2019a, 2019b). Another study evaluated the immunomodulatory and ulcer protective action of carvacrol (25, 50 & 100 mg/kg) on an animal model in which gastric lesions were made by acetic acid. Results showed carvacrol gastric healing actions and also proved that it interferes with secretion and production of inflammatory mediators in case of ulcer (Hussein et al., 2019; Silva et al., 2012; Table 1).

**TABLE 1:** Carvacrol's health benefits and method of action

No	Health perspective	Mechanism of action	References
1	Anti‐oxidant activity	Hepatoprotective effects DNA protection Anti‐oxidant activity mediates anticancer effects Increase in anti‐oxidant defense leads to improved immune system response Reduction of oxidative stress damage in the brain, liver, and kidney of rats	Alagawany et al. (2015) and Sun et al. (2020)
2	Anticancer activity	Cytotoxic, genotoxic, and proapoptotic activities with effects on cell invasion by decreasing the expression of matrix Metalloprotease 2 and 9 (melanoma cell, larynx, colon, gastric, leiomyosarcoma cells, and chronic myeloid leukemia, cells), K562, A549 non‐small‐cell lung cancer cells, MCF‐7, and MDA‐MB‐231 human metastatic breast cancer cells Genomic DNA fragmentations and caspase‐3, caspase‐6, or caspase‐9 enzymes gene expression were induced by carvacrol; also carvacrol induces apoptosis regulatory genes in human cancer and retarded growth	Zeytun and Özkorkmaz (2021)
3	Antidiabetic role	Carvacrol significantly restored PI3K/AKT signaling, which was impaired in mice with T1DM and T2DM. Carvacrol increased levels of phosphorylated PI3K, PDK1, AKT, and AS160 and inhibited PTEN phosphorylation in mice with T1DM and T2DM. Carvacrol treatment promoted GLUT4 membrane translocation in mice with T1DM and T2DM	Bayramoglu et al. (2014) and Hou et al. (2019a, 2019b)
4	Anti‐inflammatory effects	Carvacrol acts as agonist of the TRPA1 receptor Reduction in the production or release of pro‐inflammatory cytokines (TNF‐α, IL‐1β, and KC) and decrease in indicators of inflammation (MPO, NF‐κB, COX‐2) and oxidative stress (GSH, MDA, and NOx levels)	Alvarenga et al. (2016)
5	Hepatoprotective effect	Protects liver during renal injury and hepatic injury by improving liver anti‐oxidant defense and minimizing the products of lipid peroxidation Decrease TNF‐α levels in pleural lavage Decrease the levels of enzyme responsible for inducing nitric oxide synthase	Bakır et al. (2016)
6	Neuroprotective role	Carvacrol provides protection for hippocampal neurons against I/R in gerbils by inhibiting ferroptosis through increasing the expression of GPx4	Guan et al. (2019)
7	Antimicrobial	*Staphylococcus aureus*, *Staphylococcus epidermidis*, Streptococcus *Aspergillus niger*, *Aspergillus flavus*, *Alternaria alternata*, *Penicillium rubrum*, *Trichoderma viride*, Candida spp., and dermatophytes Listeria monocytogenes *Escherichia coli* O157:H7 and Salmonella *Pseudomonas aeruginosa* Interact with the cell membrane by hydrogen bonding, rendering the membranes and mitochondria more permeable and disintegrating the outer cell membrane	Alagawany et al. (2015), Gholami‐Ahangaran et al. (2022) and Sun et al. (2020)
8	Rheumatoid arthritis	Reduce arthritis signs and release of NO and IL‐17 inflammatory cytokine. Carvacrol‐mitigated LPS‐induced cell proliferation, migration, and inflammation in RA‐FLSs. The TLR4/MyD88/NF‐κB, p38, and ERK1/2 pathways might be involved in the protective effect of carvacrol	Gholijani et al. (2019) and Li et al. (2019)
9	Anti‐obesity effect	Reduce lipid accumulation during adipogenic differentiation by modulating genes associated with adipogenesis and inflammation Increase levels of very low density lipoproteins‐cholesterol (VLDL‐C), low density lipoproteins‐cholesterol (LDL‐C), and decreased level of high density lipoproteins‐cholesterol (HDL‐C)	Ezhumalai et al. (2015) and Spalletta et al. (2018)

## 4 REPRODUCTIVE ROLE

Oxidative stress decreases the number of germ cells and damages testicular tubules, as reactive oxygen species (ROS) is very important for the sperm condensation, sperm‐oocyte fusion, and hyper‐activation required for normal fertilization, but excessive lipid peroxidation and ROS could damage sperm dysfunction and cause DNA damage and loss of motility. Oxidative stress leads to the damage of spermatozoa due to the lack of cytoplasmic defensive barriers and spermatozoon plasma membranes containing polyunsaturated fatty acids (PUFA) get injured as well as destroy lipid structure in spermatozoa membranes and cause loss of motility and damage to membrane integrity. A study described by Shoorei et al. in 2019 investigated the role of carvacrol in 32 male adult Wistar rats in which diabetes was induced by streptozotocin (50 mg/kg) supplement. Findings suggested that carvacrol 75 mg/kg reduced the rate of germ cell apoptosis, reduced the activity of SOD and GPx enzymes, diminished the elevated levels of MDA, and improved morphology of the testis as well as decreased Bax and increased Bcl‐2 at the levels of gene and protein expression (Shoorei et al., 2019). A study conducted by Güvenç et al. (2019) found that carvacrol in combination with thymol has a significant impact on the quality of sperms improved by decreasing level of oxidative stress, MDA levels in testicles, liver, and kidney tissues, enhancing the GSH‐Px and catalase activities along with enhancement in spermatozoa concentration and motility (Güvenç et al., 2019). Moreover, carvacrol also improves the mean motility, movement characteristics, sperm capacitation, and fertilizing ability and prevents testicular damage (Cengİz et al., 2017). Carvacrol (25 and 50 mg/kg) prevented ketamine‐induced oxidative stress and damage in testicular tissues by lowering the level of MDA‐induced schizophrenia and increasing the anti‐oxidant enzymes (Araghi et al., 2017). Similarly, carvacrol prevents cyclophosphamide‐induced testis toxicity and damage in male rats due to its anti‐oxidative role (Cengiz et al., 2017). In adult male Sprague–Dawley rats, cisplatin induces reproductive toxicity by damaging the dermatological parameters (live sperm rate, motility, and abnormal sperm rate), increasing the oxidative stress, and inducing testicular degeneration whereas daily orally administrated carvacrol at the rate of 75 mg/kg prevented from these changes (Aksu et al., 2016).

### 4.1 Safety and toxicity

Carvacrol is considered the safest chemical compound at a low amount, approved by Federal Drug Administration (FDA), and used as a preservative in the food industry (Suntres et al., 2015).

Ghorani et al. (2021) designed a study to determine the carvacrol tolerability and safety in normal‐healthy individuals by dividing them into two groups having 1–2 mg of carvacrol per body weight a day. In the group received 1 mg/kg/day of carvacrol, their erythrocyte sedimentation rate, mean cell volume, hemoglobin, and hematocrit levels were reduced, but creatinine phosphokinase was significantly increased. Their triglyceride, phosphorus, lactate dehydrogenase, prothrombin time, mean corpuscular hemoglobin, and mean corpuscular hemoglobin concentration were significantly increased after treatment with carvacrol 1 mg/kg/day. In the group received 2 mg/kg/day of carvacrol, their high‐density lipoprotein cholesterol (HDL), total bilirubin, amylase, iron, red blood cells (RBC) count, and HCT reduced after one‐month treatment (Ghorani et al., 2021). Llana‐Ruiz‐Cabello et al. (2016) suggested that carvacrol in 81–810 mg/kg BW is safe as it does not induce toxicity of oxidative stress leading to damaging of genes and DNA investigated in vivo genotoxic study. Animals were exposed orally to 81, 256, or 810 mg carvacrol/kg body weight (BW) at 0, 24, and 45 h. Results concluded that carvacrol (81–810 mg/kg BW) is a safe dose (Llana‐Ruiz‐Cabello et al., 2016). Barnwal et al. (2018) examined swiss albino mice to see the significant role of carvacrol (25 mg/kg and 50 mg/kg) against Benzo(a)pyrene‐induced lung toxicity and inflammation. Results concluded that pretreatment with carvacrol increased the activities of anti‐oxidant enzymes and restored B (a) P‐induced lipid peroxidation (Barnwal et al., 2018).

Acute liver failure is caused by many factors such as acetaminophen overdose‐induced liver damage and leads to acute liver failure. Different doses of carvacrol with thymol (25, 50, and 100 μM) hindered the effect of APAP on inhibiting HepG2 cell growth by increasing anti‐oxidant activities and reducing pro‐inflammatory cytokines (Palabiyik et al., 2016). In a recent study, different doses of carvacrol (50 and 100 mg/kg) prevented the alcoholism‐induced liver toxicity, liver damage such as steatosis, cirrhosis, and liver fibrosis via improving the matrix metalloproteinases activities, anti‐oxidant markers, liver function, and histological changes (Khan, Bhardwaj, Shukla, Lee, et al., 2019; Khan, Bhardwaj, Shukla, Min, et al., 2019; Khan, Singh et al., 2019). Likewise, carvacrol also prevents from enhancement in.

Stojanović et al. (2018) investigated the damaging effect of carvacrol on pancreatic tissue of Wistar rats. The higher dose of carvacrol 100 and 500 mg/kg induced a significant increase in α‐amylase activity, and the 10 mg/kg dose of carvacrol prevented the increase in MDA, serum α‐amylase and lipase activities, and MDA levels in pancreatic tissues of Wistar rats (Stojanović et al., 2018). Carvacrol 25 μg/kg/day is the safest dosage to protect against Parkinson's disease induced by unilateral intraneural injection of 6‐hydroxydopamine (6‐OHDA) in adult Wistar rats analyzed to see its neuroprotective effects. Pretreatment with carvacrol for 15 days prevented the loss of DA neurons induced by 6‐OHDA in adult Wistar rats. Results concluded that anti‐oxidant, anti‐inflammatory, and pretreatment inhibited the release of IL‐1β and TNF‐α. Carvacrol also prevented the increase of mitochondrial superoxide production induced by 6‐OHDA in cultured SH‐SY5Y cells. Results indicated that carvacrol could be used as a safe drug for treating Parkinson's disease (Ribeiro et al., 2019).

Carvacrol has been proved as the safest antimicrobial thin film coating to enhance food quality and safety in food packaging and preservation. Coating of polyethylene films with an antimicrobial thin film of 225 nm thickness through a Layer‐by‐Layer (LbL) by depositing 10 bilayers of chitosan (CHI) and carvacrol loaded HNTs onto the polyethylene surface. Carvacrol‐coated films reduced the viability of a food pathogen, *Aeromonas hydrophila* by 85% and the aerobic count on chicken meat surfaces by 48% (Hussein et al., 2019; Tas et al., 2019).

## 5 CONCLUSION

The purpose of this comprehensive review was to highlight and explain the biosynthesis mechanism of carvacrol and the significant protective effects of carvacrol as an anti‐oxidant, anticancer potential, anti‐inflammatory, antimicrobial, hepatoprotective effects, and neuro‐protective and anti‐obesity effects. Scientists and researchers have studied the potential of carvacrol and thymol for cancer prevention, diabetes prevention, wound healing, and immuno‐modulatory in vivo and in vitro assays. Carvacrol exhibits a high potential for the development of new therapeutic alternatives to cure human maladies. However, the extensive studies still required to elucidate the potential therapeutic effect of carvacrol on molecular level by involving long‐term human efficacy trial with special reference to its lethal dose, toxicity, and RDA.
